# Ageism in healthcare technology: the older patients’ aspirations for improved online accessibility

**DOI:** 10.1093/jamiaopen/ooac061

**Published:** 2022-07-13

**Authors:** Dani Zoorob, Yasmin Hasbini, Katherine Chen, Victoria Wangia-Anderson, Hind Moussa, Brian Miller, Debi Brobst

**Affiliations:** Department of Obstetrics and Gynecology, University of Toledo, Toledo, Ohio 43606, USA; Department of Integrative Biosciences, Wayne State University, Detroit, Michigan 48202, USA; Department of Obstetrics and Gynecology, University of Toledo, Toledo, Ohio 43606, USA; College of Allied Sciences, University of Cincinnati, Cincinnati, Ohio 45221, USA; Department of Obstetrics and Gynecology, ProMedica Health System, Toledo, Ohio 43606, USA; Department of Informatics, ProMedica Health System, Toledo, Ohio 43604, USA; Department of Informatics, ProMedica Health System, Toledo, Ohio 43604, USA

**Keywords:** telehealth, ageism, older, access, digital exclusion

## Abstract

**Objective:**

To identify concerns, barriers and facilitators impacting the use of patient portals by older patients as well as desired features in future updates.

**Materials and Methods:**

This is a cross-sectional study consisting of 2 focus group discussions culminating in an anonymous survey administered to women who were 65 years and older receiving urogynecologic care in Northwest Ohio.

**Results:**

Of the 205 women surveyed (91% response rate), providers and healthcare systems play the primary 2 roles (73% and 69%, respectively) in facilitating patients’ use of patient portal systems and telehealth applications. Barriers to use revolved around technical difficulties (50%), privacy concerns (45%), and cost of technology (24%). The most important features desired were the ability to modify the text size within the application (47%) and an intuitive, simple interface (46%). Additional assistance for navigating technical challenges was suggested, specifically set-up of accounts (36%), saving and sharing information with caregivers (35%), and sign-in and navigation of portals (32%).

**Conclusion:**

The paucity of age-aligned medical access software and products may lead to worsening of digital exclusion and disparities in healthcare. Portal application developers and healthcare systems must advance efforts that consider the needs of those who may be older when designing patient portals.

## INTRODUCTION

The digitalization of medicine has provided healthcare systems and providers the means to deliver reliable and prompt care with notable clinical benefits overcoming barriers including servicing vulnerable populations.^1^^,^^2^ Patient portals and health applications are often the first points of entry for patients into technology-enhanced care. They provide users with an avenue for secure communication with providers, the opportunity to complete essential functions—such as booking an appointment, refilling a medication, paying a bill—quickly and effortlessly, real-time updates on labs and test results, access to physician notes, and care plans, and more.^3–5^ Patient portal applications have also evolved to include a component whereby providers can also provide direct care to the patient through telemedicine incorporated in the applications.

With a higher prevalence of multiple chronic conditions among the elderly population, patient portals and health applications provide a channel for self-management, empowerment, and autonomy.^6^^,^^7^ Unfortunately, age has been suggested as a potential limiting factor, hindering the use of technology for healthcare as well as patient portals among the elderly.^7^^,^^8^ Studies have suggested that layout and formatting, including small texts or icons, confusing navigation panes, as well as poor design in terms of colors and pictures of patient portals are critical barriers.^1^^,^^2^^,^^4^^,^^7^ Additionally, the extensive amount of information patients need to input upon signing up and logging in is perceived as a barrier.^2^^,^^6^^,^^7^^,^^9^ Moreover, research has shown that patient portals or applications have historically been challenging to print, send, or save information for sharing with caregivers, making them less likely to be used.^6^^,^^7^ Aside from portal-specific limitations, physical limitations may hinder optimal use—joint issues, hearing impairment, and vision changes, as well as psychological complaints such as computer anxiety and stress resulting from the pressure of using new technology.^2^^,^^4^^,^^6^^,^^10–13^

The increasing integration of technology in medicine with features constantly being added to the portals places the older population, who may be slower at the uptake of such innovations, at a disadvantage in their medical care. In addition to being unable to benefit specifically from the direct use of portal application components of various uses, they are also placed at a risk of receiving inadequate healthcare because of their exclusion from telemedicine services integrated at times in such portal applications. Thus, it is imperative to identify the associated difficulties for this age group and mitigate informatics-related obstacles. Furthermore, commissioners and service providers have been asked to address digital disenfranchisement resulting from internet connectivity concerns.^14^

The primary objective of our study was to identify facilitators, concerns, and barriers to patient portal use within the older female population. Secondary objectives included the identification of elderly focused enhancements while developing actionable items for developers and healthcare systems.

## MATERIALS AND METHODS

We conducted a cross-sectional study at a large academic tertiary care center in northwest Ohio, between July 1 and July 31, 2021. Our study consisted of 2 phases—focus group discussions followed by an anonymous survey with the population selected based on convenience sampling. The study was approved by the respective Institutional Review Board (Study 21-085-PHS). Inclusion criteria included age 65 and older, female gender, visual acuity permitting completion of the survey, and the ability to read and understand English.

### Focus groups

The focus groups were conducted with 10 women in 2 groups of 5. The format consisted of a semistructured interview with open-ended questions about their experience with patient portal and telehealth use. The topics of discussion addressed were developed based on an extensive literature review. The discussions were recorded, transcribed, and used to design the anonymous survey.

### Survey

The survey was administered to 225 female participants who were 65 years and older at a tertiary care center UroGynecology clinic utilizing a convenience sampling fashion. The survey consisted of 43 close-ended questions that assessed general access to technology, participant concerns regarding patient portal use, and suggested features enhancing adoption. Additionally, patient receptivity in women’s healthcare was addressed.

### Statistical analysis

Survey results were reported as descriptive statistics. Continuous variables were reported as means with standard deviation, whereas percentages were used to describe findings of true/false and agree/disagree questions. Analysis was performed using JMP software (Version 16, SAS Institute Inc., Cary, NC, 1989–2021).

## RESULTS

The survey was administered to 225 patients and achieved a 91% completion rate (Table 1). This high rate was attributed to the survey format and patients completing it during the visit downtime.

**Table 1.** Participant demographics and access to technology

Age (mean and SD in years)	68.9 (±4.9)
	Number of participants *N*, (%; total *N* = 205)
Race and ethnicity	
Caucasian race	180 (87.80)
African American race	22 (10.73)
Asian race	1 (0.49)
More than 1 race	2 (0.98)
Hispanic/Latino Ethnicity	13 (6.34)
Highest educational accomplishment	
Nonhighschool graduate	10 (4.88)
High school graduate	56 (27.32)
Junior College	14 (6.83)
Two years of college	34 (16.59)
College or undergraduate degree	47 (22.93)
Professional/postgraduate degree	44 (21.46)
Devices owned and used for portal access	
Mobile phone	194 (94.63)
Laptop computer	124 (60.49)
Tablet	113 (55.12)
Desktop computer	66 (32.20)
Other	2 (0.98)
None	4 (1.95)
Participants with continuous/reliable internet access	188 (91.71)

While potential internet access by those surveyed was noted at 91.7%, the focus group stressed that speed, reliability, and continuous availability per se disproportionally exacerbated the older population’s accessibility at the outset of the pandemic. This was explained by the limited appreciation, at the time, of such characteristics justifying the associated expenditure. The most used device for portal access was the mobile phone, with only 2% reporting having no access to any device (Table 1).

**Figure 1.**
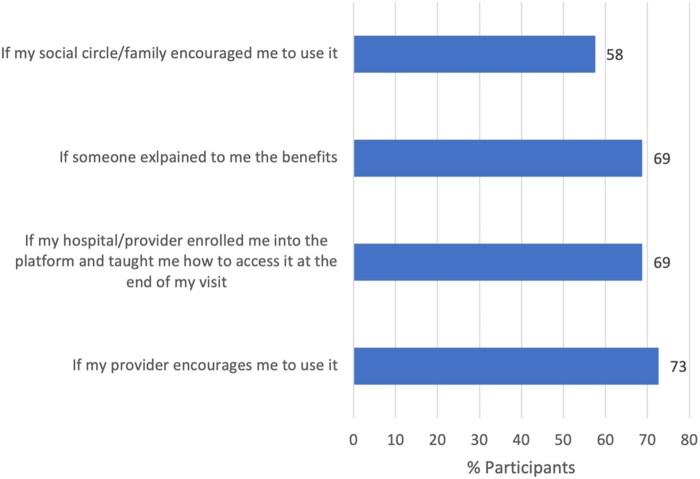
Facilitators of patient portal use among elderly. Reported figures refer to percentages of participants that reported specific facilitators enhancing their consideration of patient portals.

The majority of participants (73.0%) reported that provider encouragement would most effectively increase portal use. This was followed by a guided clarification of benefit (69%), active engagement during enrollment (69%), and endorsement by family/acquaintances (58%) (Figure 1).

Initial portal setup (36%), information sharing (35%), and sign-on (32%) are the main barriers for use of technology. Those also include typing difficulties, hurdles in log-in and navigation, small size of the text and icons, and difficulty in safely sharing medical information from the portal with caregivers such as family members.

**Figure 2.**
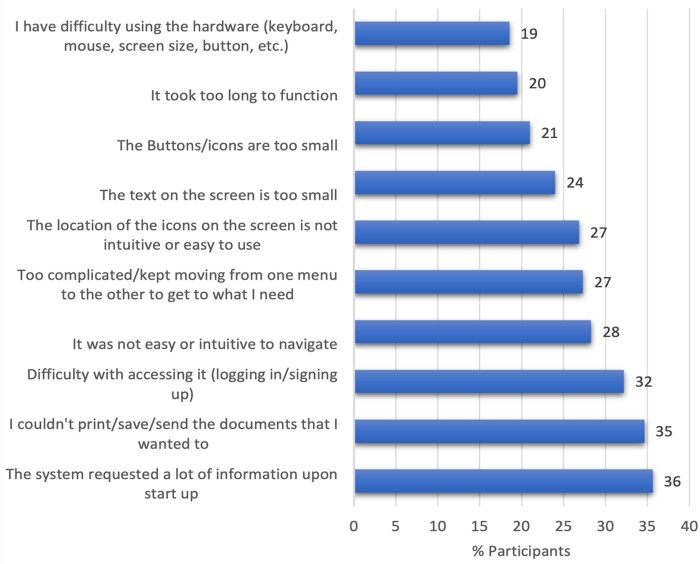
Patient-encountered technical barriers. Legend: Reported figures refer to the percentage of participants encountering personal or technical concerns during portal use.

Fear of misuse and lack of online privacy regarding personal information impacted consideration of portals in older users. Technical concerns were the primary barriers for adoption of technology (50%), followed by privacy (45%) and cost (24%) (Figure 2). The focus group stressed that there is a difference between owning a technological device and competently using it, with 17.6% of those surveyed reporting the latter.

**Figure 3.**
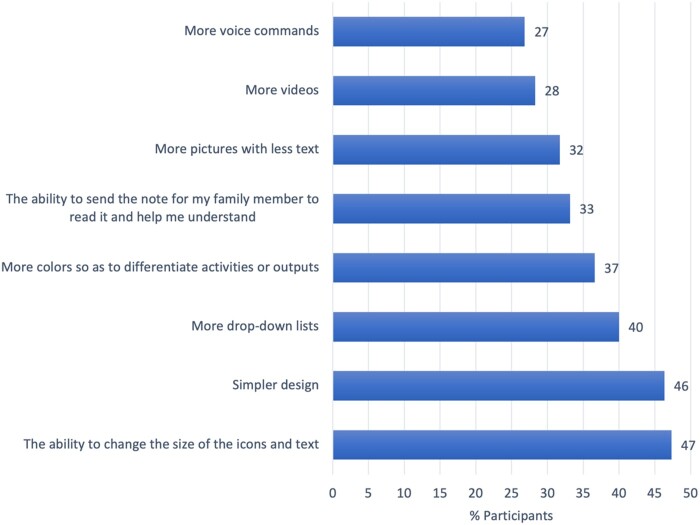
Patient-requested enhancements and features in portal updates. Figures refer to the percentage of participants that reported specific features desired to be included in patient portals.

Valued modifications requested by participants included the ability to change the size of icons and text (47%) followed by an intuitive simpler portal design (46%) (Figure 3).

Wellness-related barriers were anxiety (36%), arthritis and finger mobility/joint pain (25%), eye strain, and related physical effects (20%). Being older than 75 increased the physical constraints impacting navigation and portal use (25% compared to 12% in those <75 years).

## DISCUSSION

The key findings of our study pertain to 3 themes: facilitators and barriers to portal uptake, technical, and design concerns, as well as the perceived feasibility of use.

### Facilitators and barriers to patient portal uptake

The first factor contributing to the uptake revolves around accessibility. This includes owning a device, knowing how to use it, and having a reliable internet connection. Most of our study participants reported having access to both a device as well as reliable internet connection, which were reported as challenges among the elderly in other studies.^15^ This could be influenced by the fact that our survey sample was well-educated and historical references were prepandemic studies. Despite the high level of education in our population, patient concerns of privacy, security, technical knowledge, and hardware difficulties persisted. These concerns generate anxiety and apprehension impacting the consideration of digital medicine platforms. These need to be addressed among all the older patients, irrespective of background.

Literature has reported that most physicians are unaware of the barriers to telehealth that older patients face, including potential lack of access to internet services or to technological devices, suggesting a gap in the healthcare system.^16^ An interesting finding in our study is the vital role that providers and healthcare systems play in increasing the uptake of patient portals applications among the older patients. Studies suggest that physicians and healthcare personnel may forgo discussing available healthcare technology with those who are older due to the misperception of disinterest.^16^ Experiencing such a form of ageism leads to lower use of the internet and digital platforms for medical care.^17^^,^^18^ Providers have been shown to play a significant role in alleviating anxiety related to portals use and increased compliance when encouraged as such.^19^ Addressing the anxiety barrier attributed to new technology must be tackled by healthcare systems through development of age-appropriate educational material addressing their concerns and technical support available. Additionally, personnel must be familiarized with age-appropriate guidance.

As studies have linked smartphone ownership with the ability to obtain reliable and timely care,^20^ it is critical to address access to technology, especially among older patients. The increasing use of technology in medicine, catalyzed by the ongoing pandemic, may result in inequity in care among populations who lack online access and are impacted by digital exclusion. Policies may need to be set to ensure equal access to technology-enabled care, including devices and reliable internet, as well as that the medical staff/systems are appropriately reimbursed for such an investment.

### Technical concerns in portal design

Flexibility for the user to modify the interface was noted as an essential feature in patient portals, a survey finding which has been echoed in the literature.^2^ The diverse modifications requested indicate that the design of portals for the older patients does not exhibit a 1-size-fits-all solution. Our results correlate with other studies that suggested that the startup and sign-on processes should be simplified, the size and color of the text or icons should be modifiable, and the interface should utilize more pictures as opposed to text.^2^^,^^6^^,^^7^^,^^9^ As suggested by the literature, patients of different backgrounds and with different conditions should be involved in the design process of the patient portals to maximize adoption of perceived needs.^21^

Prioritization of the needs of such a population seem deficient in medical or in governmental policies that standardize access to technology with portal application developers and providers minimalizing efforts focused on the older population in the digitalization of medicine throughout history.^22^ The pandemic has further exposed the prevailing ageism, specifically through the rapid expansion of telehealth, where program developers prioritized the expansion to suit the majority of the population. This further stressed the marginalization of vulnerable populations such as minorities with barriers or patients who are older. This may be because digital platforms have been previously designed from the standpoint that the elderly are unable or not interested in digital medicine, further excluding them from the design process.^23^

### Perceived feasibility of use

Our study findings corroborate outcomes of other studies where practicality and ease of use are highly desirable and alleviate pressures in medical care.^4^ Some patients may perceive technology in medicine as irrelevant. Thus, it is necessary that educators reinforce the practicality and significance of digital platforms for medical care especially as telemedicine services have been shown to be helpful to the older population *per se*.^2^^,^^24^

### Strengths and limitations

Our study’s strengths include the unique 2-phased assessment where in-depth information was collected through focus group discussions followed by the anonymous survey that was completed by large cohort of patients. Our survey also included a large population with a 91% completed survey response rate, and focused specifically on technology-enabled care among older females, which has not been previously reported in the literature. However, this is not without limitations as our population consisted mainly of educated and Caucasian women. Thus, the findings may be generalizable or applicable to all patients. Additionally, it only focused on postmenopausal women and not men, where a variation in needs may be noted. However, due to the high response rate, this may have a minimal effect.

## CONCLUSIONS

Patient perceptions, identified needs, and potential barriers for embracing portal technology must become considerations critical to system optimization. The limited focus of patient portal developers, providers, and health systems on the needs of the older population may exacerbate systemic ageism. Policymakers, developers, providers, and healthcare systems must collaborate to enhance accessibility of technology-enabled care in order to offer equitable care for all.

## AUTHOR CONTRIBUTIONS

DZ: project lead, survey setup, data interpretation, and manuscript finalization; YH: data analysis, manuscript draft setup, and final approval of manuscript; KC: data analysis, manuscript draft setup, and final approval of manuscript; VWA: course mentor, project setup critical revising of manuscript, and final approval of manuscript; HM: critical revising of manuscript and final approval of manuscript; BM: concept developer, draft survey setup, and manuscript revising, final approval of manuscript; DB: project local mentor, survey revision, and project setup final approval of manuscript.

## SUPPLEMENTARY MATERIAL

Supplementary material is available at *JAMIA Open* online.

## Supplementary Material

ooac061_Supplementary_Data

## Notes

Presentations: The findings of our study were exhibited as a late-breaking podium presentation at the 2021 American Medical Informatics Association (AMIA) Annual Symposium. This was the commencing oral presentation for the session on Diversity, Equity, Inclusion, and Access to Care.
