# Supplementary material for: Ageism in healthcare technology: the older patients’ aspirations for improved online accessibility
Source: JAMIA Open. 2022 Jul 13;5(3):ooac061. doi: 10.1093/jamiaopen/ooac061 (PMC9277451; doi:10.1093/jamiaopen/ooac061)
Supplement: ooac061_Supplementary_Data [file ooac061_supplementary_data.pdf]

Ageism in Healthcare Technology - The Older Patients' Aspirations for Improved Online Accessibility

Dani Zoorob MD MHI MHA MBA, Yasmin Hasbini MD, Katherine Chen MD, Victoria Wangia-Anderson PhD MS, Hind Moussa MD MHI, Brian Miller MD, Debi Brobst MBA

Aim

- To identify concerns, barriers and facilitators impacting the use of patient portals by older patients as well as desired features in future updates.

Population

- Two groups of five women each and an anonymous survey of geriatric women receiving urogynecologic care in Northwest Ohio whose age was 65 and older
- Total of 205 surveys were completed (91% response rate)

Methods

- This is a cross-sectional study consisting of focus group discussions and anonymous surveys based on the findings

Results

- Both providers and healthcare systems play the primary roles (73%, 69% respectively) in facilitating patients' use of patient portal systems and telehealth applications.

- Barriers to use revolved around technical difficulties (50%), privacy (45%), and cost of technology (24%). The most important features desired were the ability to modify the text size within the application (47%) and an intuitive simple interface (46%).

- Additional assistance for navigating technical challenges was suggested, specifically set-up of accounts (36%), saving and sharing information with caregivers (35%), and sign-in and navigation of portals (32%).

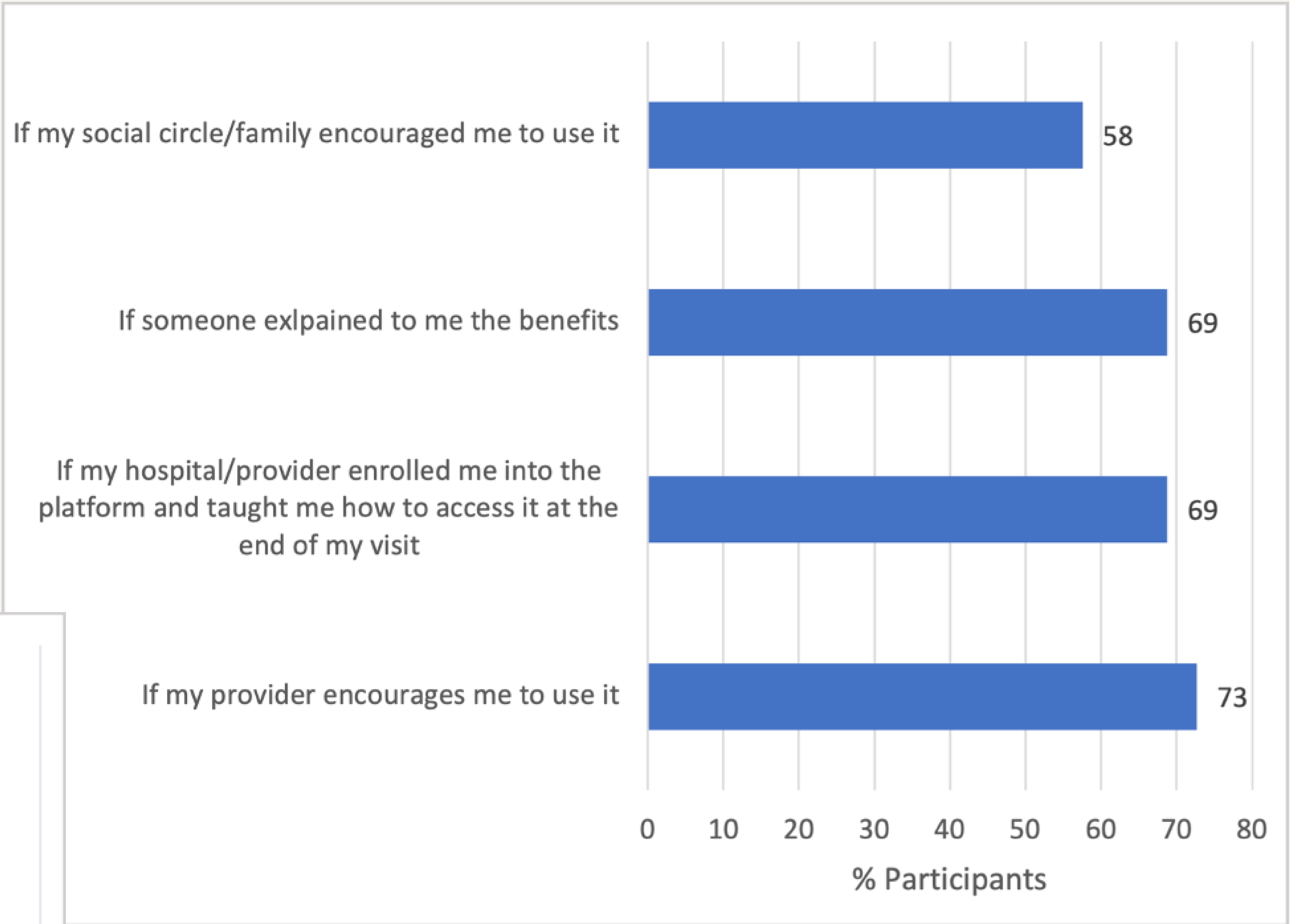

Facilitators of Patient Portal Use Among Elderly

Features Desired in Patient Portals

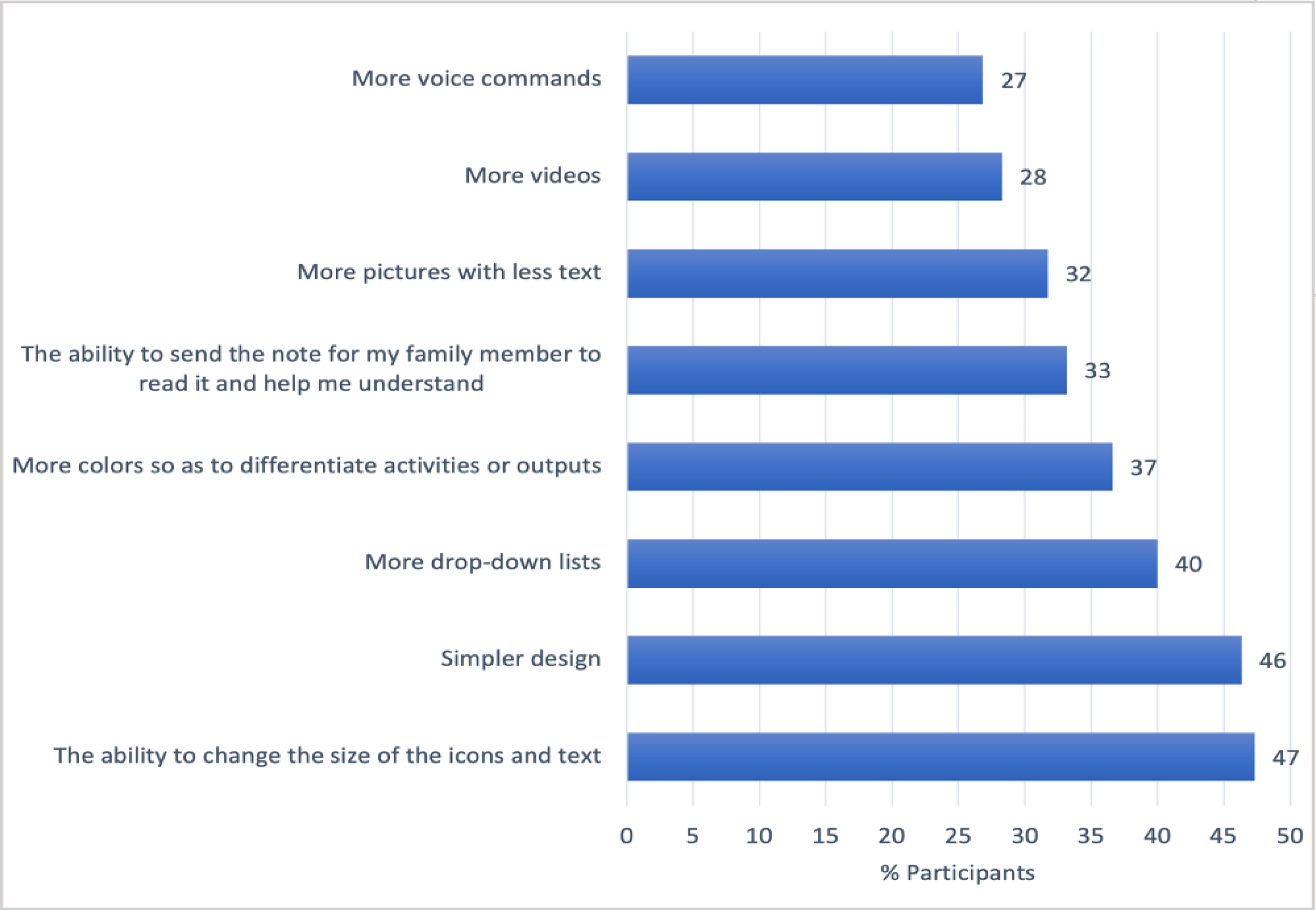

Conclusion

The paucity of age-aligned medical access software and products may lead to worsening of digital exclusion and disparities in healthcare. Portal application developers and healthcare systems must advance efforts that consider the needs of those who may be older when designing patient portals.
